# Crystal Structure of CYP3A4 Complexed with Fluorol Identifies the Substrate Access Channel as a High-Affinity Ligand Binding Site

**DOI:** 10.3390/ijms232012591

**Published:** 2022-10-20

**Authors:** Irina F. Sevrioukova

**Affiliations:** Department of Molecular Biology and Biochemistry, University of California, Irvine, CA 92697-3900, USA; sevrioui@uci.edu; Tel.: +1-949-824-1953

**Keywords:** CYP3A4, fluorol, substrate binding, crystal structure

## Abstract

Cytochrome P450 3A4 (CYP3A4) is a major human drug-metabolizing enzyme, notoriously known for its extreme substrate promiscuity, allosteric behavior, and implications in drug–drug interactions. Despite extensive investigations, the mechanism of ligand binding to CYP3A4 is not fully understood. We determined the crystal structure of CYP3A4 complexed with fluorol, a small fluorescent dye that can undergo hydroxylation. In the structure, fluorol associates to the substrate channel, well suited for the binding of planar polyaromatic molecules bearing polar groups, through which stabilizing H-bonds with the polar channel residues, such as Thr224 and Arg372, can be established. Mutagenesis, spectral, kinetic, and functional data confirmed the involvement but not strict requirement of Thr224 for the association of fluorol. Collectively, our data identify the substrate channel as a high-affinity ligand binding site and support the notion that hydrophobic ligands first dock to the nearby peripheral surface, before migrating to the channel and, subsequently, into the active site.

## 1. Introduction

Cytochromes P450 (CYPs) are the largest and most important group of enzymes involved in xenobiotic metabolism. Among 57 human CYPs, CYP3A4 is the most abundant liver and intestinal isoform that oxidizes over half of administered pharmaceuticals along with other xenobiotics, including pesticides, insecticides, herbicides, carcinogens, dietary compounds, food additives, and environmental pollutants [1,2,3]. CYP3A4 has a large and malleable active site that can accommodate chemically diverse molecules, frequently two or more at the same time. This leads to substrate promiscuity and cooperative binding that can be manifested as atypical (non-Michaelis–Menten) kinetic behavior and sigmoidal titration plots [4]. In vivo, simultaneous binding of multiple substrate/inhibitor molecules to CYP3A4 can cause serious adverse effects, such as clinically relevant drug–drug interactions and drug toxicity.

Despite the central role of CYP3A4 in drug metabolism and implications in pharmacokinetic drug interactions, there is limited understanding of how the protein adapts to a wide range of substrates and transports them from the lipid bilayer into the active site. To date, only a handful of crystal structures of CYP3A4 complexed with non-heme-ligating substrates have been reported. Bromoergocryptine (BEC) [5], midazolam [6], mibefradil, and azamulin [7] were found to bind in a productive mode, whereas erythromycin [8] and 1,7-dihydroxy-bergamottin [7] associate to the active site in a nonproductive manner, with the primary sites of metabolism remote from the catalytic center. Progesterone, in turn, docks to a peripheral hydrophobic cleft [9], thought to represent an allosteric ligand binding site. So far, there is no direct evidence that ligands can be retained inside the substrate access channel; however, as theoretical studies predict, the channel can accommodate up to two small molecules such as 7-benzyloxyquinoline [10].

**Figure 1:**
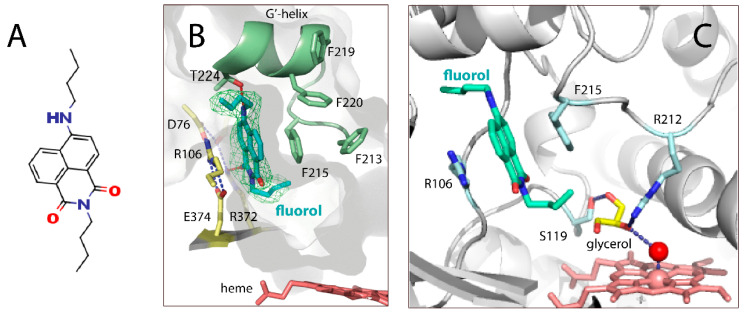
Chemical structure of fluorol (**A**) and the crystal structure of the CYP3A4–fluorol complex (**B**,**C**). (**B**) Fluorol binds inside the substrate channel, forming π–π and cation–π interactions with Phe215 and Arg372, respectively, as well as H-bonds with Thr224 and Arg372. The green mesh is a polder OMIT electron density map contoured at 3 σ level. (**C**) The Arg212 side-chain H-bonds to the heme-ligated water molecule and the nearby glycerol, also linked to Ser119.

In this study, we determined the crystal structure of CYP3A4 bound to fluorol 7GA, also known as fluorol-555 (referred to as fluorol for simplicity), and investigated their complex formation using spectral and kinetic methods. Fluorol (Figure 1A) is a small fluorescent molecule, shown to have two widely separated binding sites in CYP3A4, with the ability to act as an allosteric ligand [11,12]. The high- and low-affinity fluorol docking sites were assigned to the peripheral progesterone-binding cleft and the heme-binding pocket, respectively [12]. In the crystal structure, there is only one well-defined fluorol molecule, stacked in the substrate channel and H-bonded to Thr224 and Arg372. To verify the crystallographic ligand binding mode, spectral and kinetic measurements on the wildtype (WT) and T224A CYP3A4 were conducted. Collectively, our structural and experimental data suggest that the substrate channel could represent the high-affinity binding site for fluorol and other planar polyaromatic molecules bearing polar groups, while the peripheral cleft serves as a transient docking area.

## 2. Results

### 2.1. Crystal Structure of the CYP3A4–Fluorol Complex

Fluorol is a bright yellow chemical; thus, it was possible to monitor its co-crystallization with CYP3A4 by following the formation of orange-colored crystals. The X-ray structure of the CYP3A4–fluorol complex was solved to 2.40 Å resolution, with the R and R_free_ factors of 18.4 and 24.6, respectively (Table 1). Examination of electron density maps revealed the presence of a single, well-defined fluorol molecule bound inside the substrate access channel (Figure 1B). The fluorol ring stacks between the Phe215 ring and Arg106 guanidine (3.6–3.8 Å away), forming strong π–π and cation–π interactions, respectively. This binding mode is stabilized by two direct H-bonds with Thr224 and Arg372, established via the secondary amine and carbonyl oxygen atoms of fluorol, respectively. Hydrophobic contacts between the aliphatic tails of fluorol and the nearby residues (Phe57, Ile230, Ile223, Pro230, and Met371) further strengthen the complex. The heme is bound to a water molecule and, thus, remains in a resting low-spin, hexa-coordinated state. The water ligand H-bonds to the Arg212 guanidine which, in turn, is linked to Ser119 via polar contacts with the neighboring glycerol molecule (Figure 1C). This polar network stabilizes the F’–G’ loop (residues 208–217), which is well-defined in fluorol-bound CYP3A4 but disordered in most of the reported structures.

**Figure 2:**
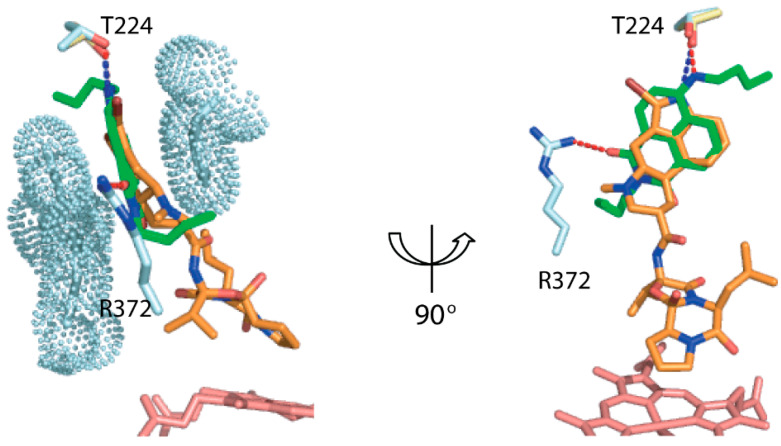
Fluorol (in green) binds to the same site in the substrate channel as the lysergic moiety of BEC (in orange; 3UA1 structure).

One striking feature was a nearly identical position of the polycyclic fluorol ring and the lysergic moiety of BEC [5], similarly sandwiched between Arg106 and Phe215, and H-bonded to Thr224 (3UA1 structure; Figure 2). Based on the T224A mutational effects, it was concluded that Thr224 assists the association and optimal orientation of BEC [5]. Therefore, we also used the available T224A mutant to validate the fluorol-bound structure of CYP3A4.

### 2.2. Effect of the T224A Mutation on Fluorol Association

#### 2.2.1. Spectral Titrations

Fluorol has limited aqueous solubility and is prone to aggregation [11]. Before setting up experiments with CYP3A4, we determined the limiting concentration of fluorol that does not lead to aggregation by monitoring emission changes. In our working solution (50 mM potassium phosphate, pH 7.4), dependence of emission intensity on fluorol concentration was linear only within the 0.1–4 μM range (Figure S1A). Addition of 0.6 mg/mL β-cyclodextrin, reported to improve solubility of fluorol [11], made no difference. For consistency, β-cyclodextrin was included in the assay buffers, while the working concentration of fluorol was limited to 3 μM to avoid/minimize artefacts associated with fluorol aggregation.

**Figure 3:**
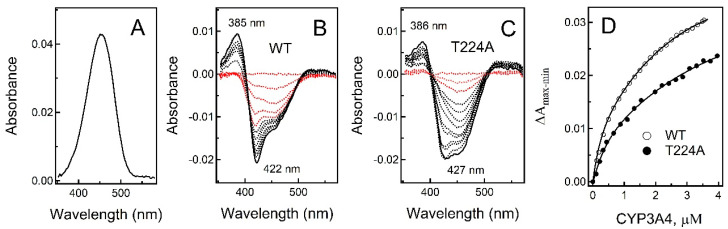
Spectral titrations of fluorol with WT and T224A CYP3A4. (**A**) Absorbance spectrum of fluorol (3 μM) with a maximum at 453 nm. (**B**,**C**) Difference absorbance spectra recorded during equilibrium titrations of fluorol with WT and T224A CYP3A4, respectively. During the first spectral phase, when [CYP3A4] < 0.5 μM, perturbations were observed only in the Soret band (red spectra). At higher [CYP3A4], a decrease in the Soret band was accompanied by formation of the 385 nm peak, indicative of the high spin transition in the heme (black spectra). (**D**) Titration plots for WT and T224 CYP3A4, built by plotting the maximal absorbance change (peak-to-trough amplitude) vs. CYP3A4 concentration. Experiments were conducted at 23 °C in 50 mM phosphate, pH 7.4, supplemented with 0.6 mg/mL β-cyclodextrin.

Because fluorol strongly absorbs in the Soret region (Figure 3A), we conducted reverse rather than direct titrations by adding small amounts of CYP3A4 to the experimental cuvette with 3 μM fluorol solution and to a buffer-containing reference cuvette. Based on the difference absorbance spectra, two spectral phases were distinguished. During the first phase, when [CYP3A4] < 0.5 μM and [fluorol]:[CYP3A4] < 6:1, spectral perturbations were detected only in the Soret band (red spectra in Figure 3B,C). The lack of changes in the 380–390 nm region implies that the heme remained in a low-spin state. At [CYP3A4] > 0.5, a decrease in the Soret absorption was accompanied by the appearance of a 385 nm peak, indicating a low-to-high spin transition in the heme. For WT CYP3A4, the first spectral phase was more pronounced, with a ~3-fold larger decrease in the Soret band, whereas the 385 nm peak formed during the second phase was ~20% higher compared to that in the T224A mutant. The difference in the total absorbance change is better seen in Figure 3D, where titration plots for WT and T224A CYP3A4 are compared. The best fittings to the binding isotherms were obtained with a two-binding-site model. The derived dissociation constants (K_d_; Table 2) allow to conclude that WT and T224A CYP3A4 have two independent binding sites for fluorol, with the affinities differing by 8–10-fold and decreasing by 25–37% when the hydroxyl group in Thr224 is eliminated.

#### 2.2.2. Fluorescence Titrations

**Figure 4:**
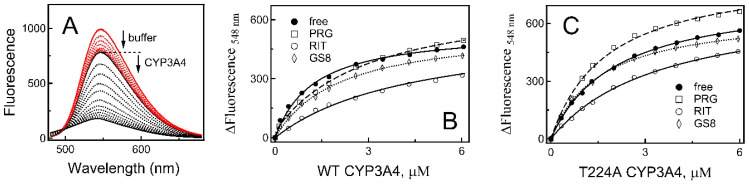
Fluorescence titrations of fluorol with WT or mutant CYP3A4. (**A**) Absolute fluorescence spectra of 1 μM fluorol. Spectra recorded after addition of small aliquots of buffer to a 1 mL sample (≤20 μL total) are shown in red. When no further changes were observed, increasing amounts of WT CYP3A4 were added (6 μM final concentration; black spectra). The respective spectra for T224A CYP3A4 were similar and are not shown. (**B**,**C**) Dependence of fluorescence changes on the concentration of WT and T224A CYP3A4, respectively, measured in the absence and presence of 20 μM progesterone (PRG), 2 μM ritonavir (RIT), or 2 μM GS8. The derived K_d_ values are listed in Table 2. Experiments were conducted at 23 °C in 50 mM phosphate, pH 7.4, supplemented with 0.6 mg/mL β-cyclodextrin.

Equilibrium fluorescence titrations were used as an alternative approach for K_d_ determination. One advantage of fluorol is a large Stokes shift (difference between the absorption and emission maxima; Figure S1B) and sensitivity to environment, as emission becomes more intense and blue-shifted when polarity of the medium decreases (Figure S1C). Moreover, spectral overlap between the emission band of fluorol and Q-bands of CYP3A4 enables fluorescence resonance energy transfer (FRET) to the heme [12]. This process dominates during complex formation with CYP3A4, because emission is decreased rather than enhanced when fluorol transfers from an aqueous media to a more hydrophobic protein environment. One complication that we encountered during fluorescence measurements was a nonspecific decrease in emission, presumably due to adhesion of fluorol to plastic pipette tips. To overcome this problem, before each titration, small amounts of buffer were added to fluorol solution in 1–2 μL increments (8–10 additions; <2% of volume change) and mixed using the same low-retention pipette tip. When no further changes in fluorescence intensity were observed, the same tip was used for addition and mixing of CYP3A4 aliquots (Figure 4A). The resulting binding isotherms (Figure 4B,C) confirmed the existence of two independent fluorol binding sites in WT CYP3A4, with the K_d_ values comparable to those derived from spectrophotometric titrations (Table 2). In contrast, only a low-affinity site was detected in the T224A mutant, where fluorescence decay was ~20% larger. This means that the low-affinity site in T224A CYP3A4 is less solvated and/or lies closer to the heme.

To locate the docking sites, fluorescence changes were monitored in the presence of progesterone, ritonavir, or GS8. There is structural and experimental evidence that progesterone preferably binds to the peripheral surface in the F’–G’ helix/loop region (Figure S2A) [9,13,14]. Two latter compounds are strong CYP3A4 inhibitors that directly ligate to the heme (K_d_ of 50 and 160 nM, respectively) [15,16]. Ritonavir’s isopropyl-thiazole end-group occupies most of the substrate channel [16], which could prevent association of fluorol (Figure S2B). GS8 is an analogue of ritonavir with a shorter and more flexible aliphatic end-group [15] that only partially fills the substrate channel. As a result, in GS8-bound CYP3A4, fluorol might still be able to bind to the channel, albeit in a different orientation (Figure S2C). Indeed, as seen from titration plots (Figure 4B,C), emission quenching was significantly reduced when fluorol was mixed with ritonavir-bound WT CYP3A4 and only moderately for the GS8-bound form (by 30% and 10%, respectively). The smallest changes were observed in the presence of progesterone. For the T224A mutant, ligand-dependent differences in emission were bidirectional: 20% and 7% decreases for the ritonavir- and GS8-bound forms, respectively, and 20% increase in the presence of progesterone. In all cases, the best fittings were obtained with a one-site model (K_d_ values are listed in Table 2). Thus, regardless of what ligand is bound, only the low-affinity site was available for fluorol in both WT and T224A CYP3A4.

#### 2.2.3. Fluorol Binding Kinetics

**Figure 5:**
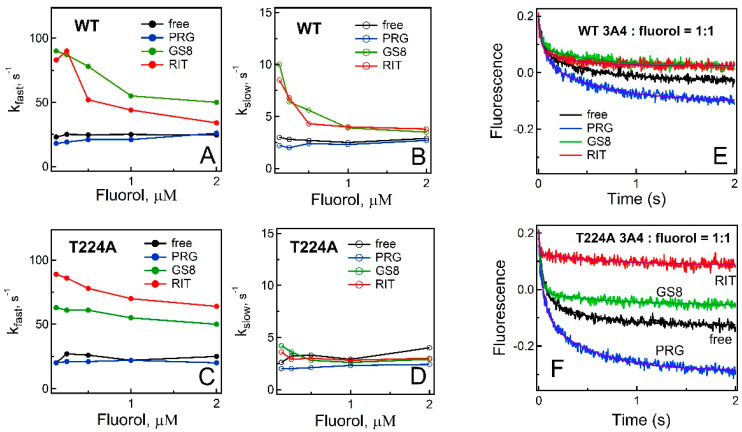
Fluorol binding kinetics. Dependence of rate constants for the fast and slow phases (*k*_fast_ and *k*_slow_, respectively) of fluorol association to 1 μM WT (**A**,**B**) and T224A CYP3A4 (**C**,**D**) on fluorol concentration. (**E,F**) Kinetic traces recorded upon mixing 2 μM fluorol with an equimolar amount of WT or T224A CYP3A4, respectively, in the absence and presence of 20 μM progesterone (PRG) or 2 μM inhibitors, GS8 or ritonavir (RIT). Fluorescence changes were monitored at 23 °C in 50 mM phosphate, pH 7.4, supplemented with 0.6 mg/mL β-cyclodextrin, with λ_ex_ of 455 nm and 495 nm cutoff emission filter. Solid lines are fitting curves.

During equilibrium titrations, fluorescence spectra were usually recorded 10–15 min after addition of CYP3A4, which could allow fluorol to adjust/change its initial binding position. To better understand the substrate/inhibitor effects, the kinetics of CYP3A4–fluorol complex formation were measured by monitoring decay of fluorol emission in a stopped-flow spectrophotometer. Before experiments with CYP3A4, control measurements were performed, where fluorol was mixed with buffer in the absence or presence of progesterone, ritonavir, or GS8. Neither ligand was found to perturb the fluorescent properties of fluorol (Figure S3), which ruled out formation of nonspecific ligand–ligand interactions. Fluorol binding to WT and T224A CYP3A4 was biphasic within the studied time interval (2 s). The rate constants for the fast and slow phases (*k*_fast_ and *k*_slow_, respectively) determined for ligand-free and progesterone-bound CYP3A4 were virtually independent on fluorol concentration (Figure 5A–D). In contrast, *k*_fast_ was enhanced for the ritonavir- and GS8-bound forms, most significantly at sub-equimolar concentrations of fluorol (by threefold). Likewise, *k*_slow_ was elevated for ritonavir- and GS8-bound WT CYP3A4 but remained unchanged for the T224A mutant.

The amplitude of quenching was another parameter markedly altered by the mutation. Kinetic traces recorded after mixing equimolar amounts of CYP3A4 and fluorol are shown in Figure 5E,F. For WT, changes in the amplitude were moderate and bidirectional: a ~20% decrease in the presence of ritonavir and GS8, and a ~40% increase in the presence of progesterone (Figure 5E). For T224A CYP3A4, changes in the amplitude were also bidirectional but more pronounced (Figure 5F): larger by 35–50% for the ligand-free and progesterone/GS8-bound protein and 60% smaller for the ritonavir-bound form.

FRET efficiency is reversely proportional to the donor–acceptor distance and, hence, it is possible to deduce where fluorol may dock based on relative changes in fluorescence quenching. In the inhibitor-bound WT CYP3A4, the active site is fully occupied, while the channel is partially or mostly accessible. The fact that fluorol emission is quenched equally in ritonavir- and GS8-bound CYP3A4 and to a lower extent than in the ligand-free protein suggests that fluorol is forced to dock to a more solvated and/or remote site, e.g., at the entrance (distal side) of the substrate channel. Progesterone has the opposite effect, meaning that fluorol associates to a less solvated area and/or closer to the heme. According to the quenching kinetics, fluorol docks to the same area in ligand-free T224A CYP3A4 (compare blue trace in Figure 5E and black trace in Figure 5F). Considering the mutational and ligand-dependent effects, the most probable binding site for fluorol would be at the proximal end of the substrate channel or within the catalytic cavity.

Summing up, our kinetic and equilibrium titration data allow to conclude that (i) the high-affinity fluorol binding area lies within the substrate channel and could coincide with the crystallographic binding site, (ii) association to the intra-channel site is assisted by direct H-bonding to Thr224 and, when it is eliminated, fluorol moves closer to the heme, and (iii) the lower affinity area, serving as a transient binding site, likely overlaps with the progesterone docking surface, and, when it is occupied, fluorol is directed straight into the channel, sliding deeper and closer to the active site.

### 2.3. Fluorol Metabolism

**Figure 6:**
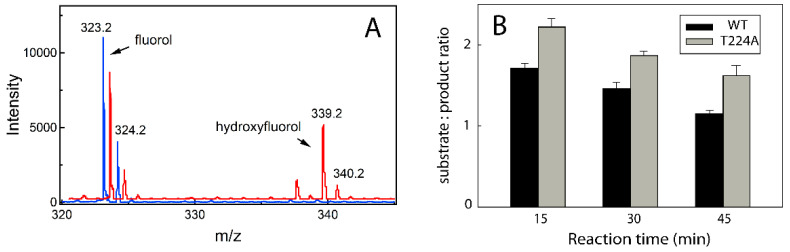
CYP3A4-dependent oxidation of fluorol. (**A**) Mass spectra of the reaction mixture before and after 15 min incubation with NADPH (in blue and red, respectively). The oxidation product of fluorol has a mass shift of +16 Da, suggesting a single-site hydroxylation. (**B**) Time-dependent changes in the substrate/product ratio for the reaction catalyzed by WT and T224A CYP3A4.

The ability of fluorol to approach and modulate the heme coordination environment suggests that it may undergo enzymatic oxidation. This possibility was tested in a reconstituted system with cytochrome P450 reductase. Mass spectrometry analysis showed that a single metabolite at m/z 339.2 was formed in a NADPH-dependent manner (Figure 6A). The mass shift of +16 Da implies that fluorol undergoes a single-site oxidation. We did not attempt to identify the site of metabolism but measured time-dependent changes in the substrate/product ratio for WT and T224A CYP3A4. As seen from Figure 6B and Figure S4, the mutant was capable of oxidizing fluorol but ~20% less efficiently. This supports the structural and spectral data and further indicates that the polar interaction with Thr224 assists but is not critical for the binding of fluorol.

## 3. Discussion

CYP3A4 is the major and most clinically relevant drug-metabolizing enzyme in the human body. Compared to other CYPs, CYP3A4 has a larger and more flexible active site and an extended substrate channel. One wall of the channel, formed by the β1 sheet, is flat and rigid and stabilized by two strong salt bridges, Asp76–Arg106 and Asp76–Arg372 [17]. The opposite side is formed by the highly flexible and Phe-rich F’–G’ fragment, which contains four out of seven Phe residues comprising the Phe cluster, a unique structural element thought to regulate substrate recognition/specificity and allosteric behavior of CYP3A4 through modulation of the shape, size, and accessibility of the substrate binding pocket [6,9,18,19]. Transfer of hydrophobic substrates from the lipid bilayer into the catalytic cavity may also be facilitated by the Phe cluster [9,20]. Structural and experimental data presented here provide additional insights on how this transition may occur.

According to the crystal structure, the complex with fluorol is stabilized through aromatic stacking and cation–π interactions and via direct H-bonding to two channel residues from the opposite walls, Thr224 and Arg372 (Figure 1B). We did not attempt to eliminate the H-bonding ability of Arg372 due to its engagement in the extensive polar network that helps maintain structural integrity of CYP3A4. Thr224, on the other hand, resides at the entrance of the substrate channel, and its Ala substitution has no effect on the folding and heme incorporation in CYP3A4 [5]. Therefore, we used the available T224A variant to validate the structure by testing whether residue 224 assists complex formation with fluorol or not. One complicating issue was the high tendency of fluorol to self-aggregation in aqueous solutions. To avoid or minimize this undesired effect, the maximal working concentration of fluorol was limited to 3 μM (Figure S1C). Even with this limitation, it was possible to make several important observations. First, we showed by absorbance and fluorescence spectroscopy that WT CYP3A4 has two binding sites for fluorol, differing in affinity by ~8–10-fold (Table 2). For the T224A variant, the two-site binding was detected only by absorbance spectroscopy. The fact that the magnitude of absorbance changes corresponding to occupation of the high-affinity site was twofold smaller in the mutant (Table 2) suggests that Thr224 comprises the high-affinity site or is situated nearby. The T224A mutation modulated the binding kinetics and ligand-dependent spin transition as well.

**Figure 7:**
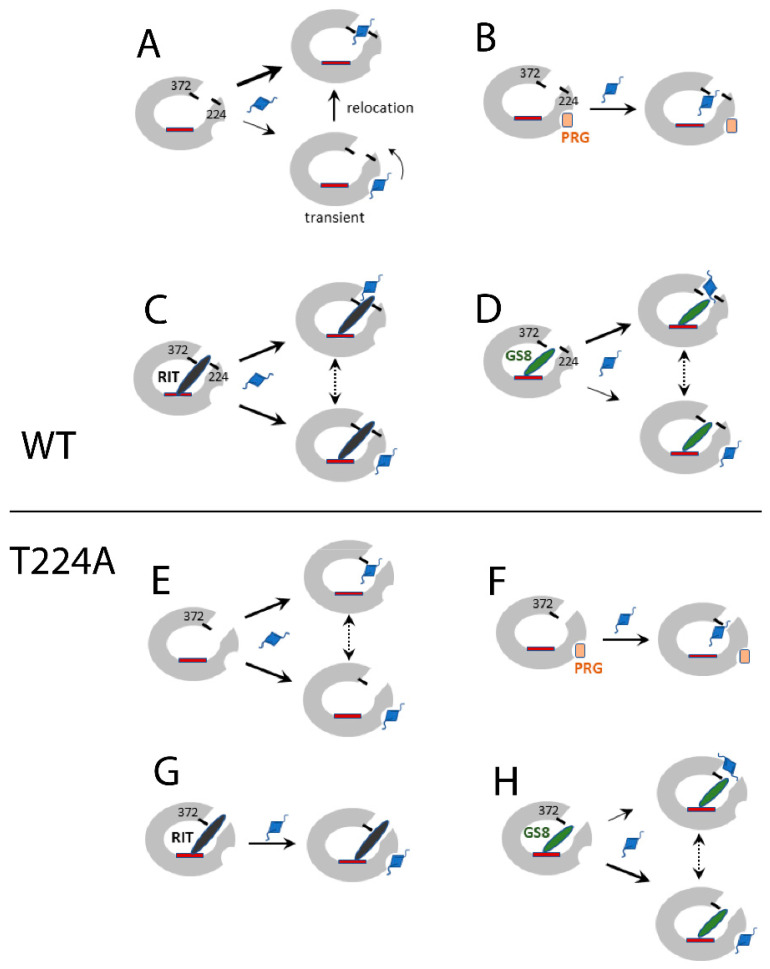
Proposed fluorol binding process. (**A**,**E**) In ligand-free WT, fluorol may transiently associate to the peripheral area then migrate to the preferable high-affinity intra-channel site by anchoring to Thr224 and Arg372 through H-bonds. In T224A CYP3A4, affinity for the intra-channel site is decreased, owing to which fluorol binds in an altered orientation or remains at the peripheral site. (**B**,**F**) Progesterone (PRG) occupies the peripheral site and changes the channel conformation. As a result, fluorol inserts into the channel differently, somewhat closer to the heme, and slides deeper into the channel/cavity when Thr224 is eliminated. (**C**,**G**), Ritonavir (RIT) blocks the active site and most of the channel. Nevertheless, in WT CYP3A4, fluorol can dock at the entrance of the channel and at the peripheral site but only to the latter site in the mutant. (**D**,**H**), The shorter GS8 leaves a larger part of the channel accessible, allowing fluorol to dock, albeit in a distinct orientation. Alternatively, fluorol can bind to the peripheral surface, which is the preferable site upon Thr224 elimination.

To get further insights into possible fluorol docking/relocation sites after the hydroxyl removal in Thr224, we assessed changes in the equilibrium and transient quenching in the presence of ligands with the known binding modes (Figure 4, Figure 5 and Figure S2). Based on our results and previously reported data [12], we propose the following chain of events for the fluorol binding process (Figure 7): in WT CYP3A4, when no other ligands are bound, fluorol associates to the high-affinity intra-channel site directly or after relocation from the peripheral site, serving as a transient docking area (Figure 7A). In the mutant, affinity for the intra-channel site is reduced and, thus, fluorol would bind to this or the peripheral site without preference (Figure 7E). In the presence of progesterone, the peripheral site is occupied, and fluorol associates solely to the intra-channel site in both the WT and the mutant (Figure 7B,F). However, progesterone seems to alter the channel conformation, forcing fluorol to bind in a different orientation, closer to the heme, and to slide deeper into the channel or catalytic cavity when Thr224 is eliminated. Ritonavir fully blocks the active site and most of the substrate channel and cannot be displaced by smaller/weaker ligands such as fluorol. Therefore, in ritonavir-bound WT CYP3A4, fluorol associates either at the entrance (distal end) of the channel or at the peripheral site. In the mutant, the latter site is preferable, if not exclusive (Figure 7C,G). In GS8-bound CYP3A4, the large portion of the channel remains accessible and, hence, fluorol can associate to the distal end of the channel, albeit in a distinct orientation, or dock to the peripheral site (Figure 7D). In the mutant, the latter area is a preferable binding area (Figure 7H).

According to our study, there are at least two areas that have lower affinity for fluorol than the intra-channel site. One is the peripheral site, which coincides or overlaps with the progesterone binding area, while another lies within the catalytic cavity, where fluorol transports to undergo oxidation. Due to a narrow range of fluorol concentrations used, the low-affinity site could not be saturated. Thus, it is unclear the occupation of which site leads to a high-spin transition. The prior study, where up to 60 μM fluorol was used, identified the peripheral area as a high-affinity site and the catalytic cavity as a low-affinity area (K_d_ of ~3 μM and 10 μM, respectively). Furthermore, it was concluded that the high-spin transition occurs when fluorol associates to the peripheral site, while the binding of the second ligand either partially reverses or does not affect the high-spin shift [12]. Under our conditions, no spin reversal or cooperativity in fluorol binding were observed, as the absorbance and fluorescence isotherms were hyperbolic rather than sigmoidal (Figure 3D and Figure 4B,C). This also contradicts the prior study, which reported strong cooperative interaction between fluorol molecules based on the Hill coefficient of 1.93 [12]. Again, this could be due to differences in the concentration range and/or because of fluorol aggregation.

**Figure 8:**
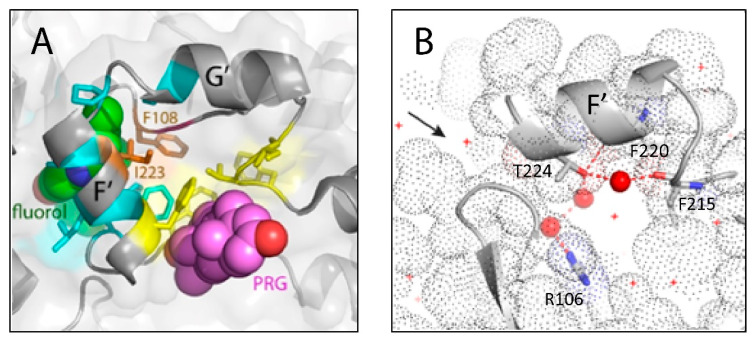
(**A**) Proximity of the peripheral and crystallographic ligand-binding sites. Residues lining the fluorol binding site are in cyan and orange; those predicted by the earlier study [12] are in yellow and orange. Fluorol and progesterone (PRG) are in CPK representation and colored in green and magenta, respectively. (**B**) Thr224 forms direct and water-mediated H-bonds with the main chain atoms of Phe215 from the F–F’ loop, Phe220 from the F’ helix, and the side-group of Arg106 from the opposite wall of the substrate channel (5VCC structure). H-bonds are shown as red dotted lines; red spheres and crosses are water molecules. Entrance into the substrate channel is indicated by an arrow.

Importantly, the intra-channel and peripheral sites lie in proximity (Figure 8A) and, hence, can be conformationally inter-dependent, in that structural changes at one site can induce reorganization at another site. This possibility is supported by accelerated MD simulations, which showed that testosterone but not progesterone could migrate directly from the peripheral site into the substrate channel by disrupting the Phe cluster [20]. Whether fluorol could do the same or must dissociate from the peripheral site to reach the channel remains to be established. Regardless, the proximity, high mobility, and mutual interdependence of the intra-channel and peripheral sites could reconcile our and the previous results on CYP3A4–fluorol interaction. According to FRET measurements for pyrene-labeled CYP3A4 [12], the high-affinity fluorol binding area was located near residues 108, 109, 213, 219, 223, and 238–241, leading to a conclusion that the high-affinity site coincides with the progesterone binding area (Figure 8A). However, two of these residues, Phe108 and Ile223, comprise the crystallographic but not peripheral site. Thus, it would be challenging, if possible, to differentiate between the neighboring sites solely based on fluorescence quenching. Even so, the residue overlap and the fact that the F’/G’ helices and B–C loop lining the peripheral site remain dynamic when the ligand is bound to CYP3A4 [20,21] support the possibility that fluorol initially binds to the peripheral area and then migrates directly into the channel.

It is intriguing that none of the prior computational studies identified Thr224 as residue that could facilitate the ligand association to CYP3A4 [17,20,22,23,24,25,26,27]. The CYP3A4–fluorol complex is the fourth structure where the ligand binding mode is stabilized via direct H-bonding to Thr224 [5,28]. Thus, participation of Thr224 in the ligand binding process could be a more general rule rather than an exception. The high-resolution structure of ligand-free CYP3A4 (PDB ID 5VCC) shows that Thr224 is linked directly or via water mediated H-bonding contacts to the main/side-chain atoms of Phe215, Phe220, and Arg106 from the F–F’ loop, F’ helix, and β1 sheet, respectively (Figure 8B). By disrupting this polar network, the T224A mutation could alter the folding and mobility of the F’/G’ helices, thereby affecting conformational dynamics of both the peripheral and intra-channel sites. Further supporting evidence for the interdependence of these sites is alteration of spin transition, S_50_ (concentration at half saturation), and the Hill coefficient for progesterone caused by the T224A mutation (Figure S5).

In conclusion, the newly determined crystal structure of CYP3A4 complexed with fluorol identified the substrate access channel as a high-affinity ligand docking area, well suited for the binding of planar polyaromatic molecules bearing polar groups, through which H-bonds with the channel residues, such as Thr224 and Arg106, can be established. The Thr224-to-Ala substitution did not preclude the binding of fluorol but altered its association kinetics, spin transition, and enzymatic oxidation. Collectively, our data lead to better understanding of the ligand binding process and suggest that fluorol docks first to the peripheral crevice outside the F’-G’ helix/loop region, before migrating to the higher affinity intra-channel site and, subsequently, into the catalytic cavity. Further studies are warranted to determine the interplay between the peripheral and intra-channel sites, and whether the latter area can serve as a regulatory allosteric site.

## 4. Materials and Methods

*Protein Expression and Purification*—Human Δ3–22 CYP3A4 and the T224A variant were produced as reported previously [29,30].

*Spectral Titrations—*Equilibrium ligand binding to CYP3A4 was monitored using a Cary 300 spectrophotometer at 23 °C in 50 mM phosphate, pH 7.4, containing 0.6 mg/mL β-cyclodextrin (Santa Cruz Biotechnology, Dallas, TX, USA), using a 10 mm light path cuvette. Ten millimolar stock of fluorol (2-butyl-6-(butylamino)-1*H*-benzo[de]isoquinoline-1,3(2*H*)-dione; Exciton, Dayton, OH, USA) dissolved in methanol (ε_440 nm_ = 14.0 mM^−1^·cm^−1^) [31] was diluted with DMSO to prepare 1–3 mM working solutions. During spectral titrations, small aliquots of WT or T224A CYP3A4 were added to the experimental cuvette with a 3 μM fluorol solution (made from 1 mM stock in DMSO) and to the reference cuvette that contained buffer and the same amount of solvent. Difference absorbance spectra were recorded 5–10 min after fluorol addition, when no further spectral perturbations could be detected. The observed absorbance changes (peak-to-trough difference) were plotted vs. fluorol concentration and fitted using a nonlinear regression analysis to derive dissociation constants.

*Fluorescence measurements—*Equilibrium fluorescence titrations were conducted on a Hitachi F-7000 fluorescence spectrophotometer at 23 °C in 50 mM phosphate buffer, pH 7.4, containing 0.6 mg/mL β-cyclodextrin. Fluorol solution (1 μM) was first titrated with small amounts of buffer using a low-retention plastic pipette tip until no further changes in fluorescence were detected (λ_ex_ = 440 nm); then, the same tip was used for CYP3A4 additions (6 μM final concentration). This experiment was repeated in the presence of 20 μM progesterone, 0.4 μM ritonavir, or 0.4 μM GS8. CYP3A4-dependent changes in fluorescence intensity were plotted vs. CYP3A4 concentration, and K_d_ values were derived from nonlinear regression fittings.

Kinetics of CYP3A4–fluorol complex formation were measured in a SX.18MV stopped-flow apparatus (Applied Photophysics, Leatherhead, UK) at 23 °C in 50 mM phosphate buffer, pH 7.4, containing 0.6 mg/mL β-cyclodextrin. WT or T224A CYP3A4 (2 μM) was mixed with 0.25–4 μM fluorol solution prepared from 1–3 mM stocks in DMSO. Equal amounts of DMSO were added to the protein solutions to avoid buffer-related changes in fluorol emission. Fluorescence changes were monitored with λ_ex_ of 455 nm and a 495 nm cutoff emission filter. Experiments were repeated in the presence of 20 μM progesterone (K_d_ for the high- and low-affinity sites is 5 and 45 μM, respectively; our estimate), 2 μM ritonavir, or GS8 (K_d_ of 0.05 μM and 0.16 μM, respectively) [15,16]. Kinetics were analyzed using the Igor Pro software (WaveMetrics, Oswego, OR, USA).

*Fluorol metabolism—*Oxidation of fluorol by WT and T224A CYP3A4 was assessed in a reconstituted system with cytochrome P450 reductase. Reaction was carried out at 37 °C in 100 mM potassium phosphate, pH 7.4, containing catalase and superoxide dismutase (2 units/mL each), 1 μM WT or T224A CYP3A4, 2 μM CPR, NADPH regenerating system (10 mM glucose, 0.2 mM NADP^+^, and 2 units/mL glucose-6-phosphate dehydrogenase), and 25 μM fluorol. After addition of 80 μM NADPH, samples were gently shaken, and 0.5 mL aliquots were taken in 15 min intervals. The reaction was stopped by adding 2 mL of dichloromethane. After 30 s vortexing, 1 mL of 0.3 M NaCl was added, and the sample was vortexed again and centrifuged for 10 min at 3000× *g* and 4 °C. The bottom organic phase (1 mL) was transferred to a glass vial, evaporated under the stream of nitrogen, and dissolved in 150 μL of methanol. Samples were analyzed by electrospray ionization mass spectroscopy using a Micromass LCP Premier mass spectrometer (Waters, Milford, MA, USA) in the negative ion mode, with 0.2 mL/min methanol flow. Chromatogram analysis and integrations were performed with Waters MassLynx MS software.

*Crystallization of the CYP3A4-fluorol complex—*WT CYP3A4 (100 mg/mL) in 100 mM phosphate buffer, pH 7.4, 20% glycerol, and 100 mM NaCl was mixed with a sixfold excess of fluorol from a freshly prepared 50 mM solution in DMSO. The mixture was centrifuged to remove the precipitate. The supernatant (0.5 μL) was mixed with an equal volume of solution #23 from the Hampton Research PegIon2 kit (0.1 M DL-malic acid pH 7.0, 12% polyethylene 3350 glycol) and equilibrated against the same solution using a sitting drop vapor diffusion method. The next day, orange-colored crystals were harvested, cryoprotected with Paratone-N oil, and frozen in liquid nitrogen.

*Determination of the X-ray Structure—*X-ray diffraction data were collected at the Stanford Synchrotron Radiation Lightsource beamline 11-1. The crystal structure was solved by molecular replacement with PHASER [32] and 1TQN as a search model. The ligand was built with eLBOW [33] and manually fit into the density with COOT [34]. The initial model was rebuilt and refined with COOT and PHENIX [33]. The polder OMIT electron density map was calculated using PHENIX. Data collection and refinement statistics are summarized in Table 1. The atomic coordinates and structure factors for the fluorol-CYP3A4 complex were deposited in the Protein Data Bank with the ID code 8DYC.

## Figures and Tables

**Table 1:** Data collection and refinement statistics.

*Data Statistics*	
Space group	I222
Unit cell parameters	*a* = 78 Å, *b* = 101 Å, *c* = 130 Å; α, β, γ = 90
Molecules per asymmetric unit	1
Resolution range (Å)	71.91–2.40 (2.53–2.40) ^a^
Total reflections	90,908 (13,973)
Unique reflections	20,233 (2963)
Redundancy	4.5 (4.7)
Completeness	98.8 (99.7)
Average *I*/*σI*	7.6 (1.1)
R_pim_	0.042 (0.878)
CC ½	0.998 (0.362)
*Refinement statistics*	
*R*/*R*_free_^b^	18.4/24.6
Number of atoms:	
Protein	3734
Solvent	6
R.m.s. deviations:	
Bond lengths, Å	0.013
Bond angles, °	1.743
Wilson B-factor, Å^2^	67
Average B-factor, Å^2^:	
Protein	85
Ligand	94
Solvent	68
Ramachandran plot ^c^ (residues; %)	
Preferred	436 (95%)
Allowed	23 (5%)
Outliers	0

^a^ Values in brackets are for the highest-resolution shell. ^b^ *R*_free_ was calculated from a subset of 5% of the data that were excluded during refinement. ^c^ Analyzed with PROCHECK.

**Table 2:** Effect of the T224A mutation and other CYP3A4 ligands on the binding affinity of fluorol.

	K_d_, μM	
	*Spectrophotometric ^a^*	*Fluorometric ^b^*
	*WT CYP3A4*	
Free	0.50 ± 0.04 ^c^ (28%) ^e^	0.15 ± 0.03 (25%)
	4.2 ± 0.5 ^d^	2.4 ± 0.4
20 μM PRG	ND ^f^	2.4 ± 0.2
0.4 μM RIT	ND	4.8 ± 0.6
0.4 μM GS8	ND	2.1 ± 0.3
		*T224A CYP3A4*
Free	0.67 ± 0.12 (14%)	2.0 ± 0.3
	6.6 ± 0.8	
20 μM PRG	ND	1.8 ± 0.2
0.4 μM RIT	ND	3.5 ± 0.3
0.4 μM GS8	ND	1.6 ± 0.2

^a, b^ Determined from titration plots shown in Figure 3D and Figure 4B,C. ^c, d^ K_d_ values for the high- and low-affinity sites, respectively. ^e^ Percentage of the absorbance or fluorescence change due to the binding of fluorol to the high-affinity site. ^f^ Not determined. All values are the average of three independent measurements ± standard deviation.

## Data Availability

Crystallographic data presented in this study are publicly available in the Protein Data Bank with the ID code 8DYC.

## Supplementary Materials

The following supporting information can be downloaded at: https://www.mdpi.com/article/10.3390/ijms232012591/s1.

## Abbreviations

CYP3A4	Cytochrome P450 3A4
BEC	Bromoergocryptine
WT	Wildtype
