# Supplementary material for: Crystal Structure of CYP3A4 Complexed with Fluorol Identifies the Substrate Access Channel as a High-Affinity Ligand Binding Site
Source: Int J Mol Sci. 2022 Oct 20;23(20):12591. doi: 10.3390/ijms232012591 (PMC9604483; doi:10.3390/ijms232012591)
Supplement: Supplementary file 1 [file ijms-23-12591-s001.zip › ijms-1984511-supplementary.pdf]

Crystal structure of CYP3A4 complexed with fluorol identifies the substrate access channel as a high affinity ligand binding site

Irina F. Sevrioukova

SUPPLEMENTARY MATERIALS

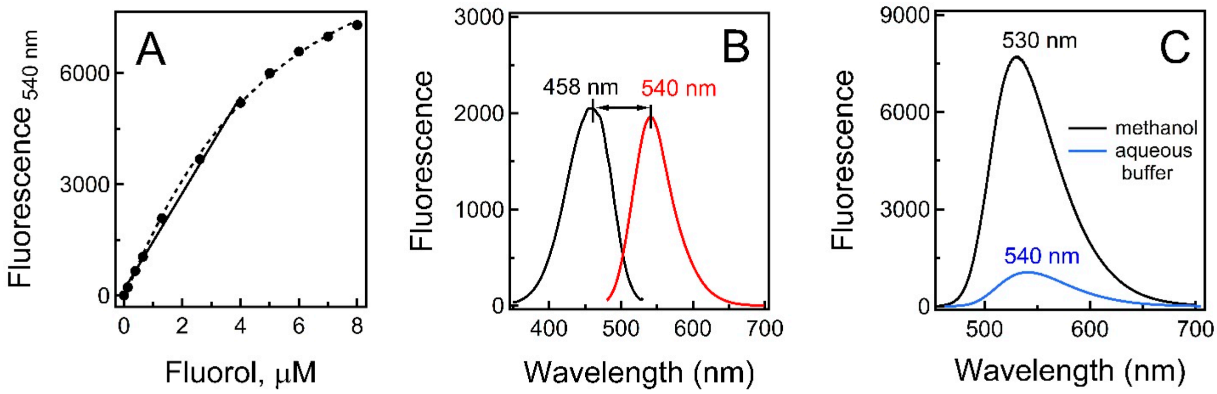

Figure S1. Fluorescent properties of fluorol. **A**, Emission of fluorol at increasing concentrations. In 50 mM potassium phosphate buffer, pH 7.4, linearity was observed only within the 0.1-4 μM range regardless of whether β-cyclodextrin (0.6 mg/ml) was present or not. **B**, Excitation and emission spectra of 2 μM fluorol in 50 mM phosphate, pH 7.4 (in black and red, respectively). The absorbance spectrum is identical to the excitation spectrum. The Stokes radius for fluorol, λ_{em} - λ_{abs}, is 82 nm. **C**, Emission of 1 μM fluorol in methanol and in 50 mM phosphate buffer, pH 7.4. In a hydrophobic environment, fluorescence intensity increases by multi-fold, while the peak maximum shifts by 10 nm toward the shorter wavelengths.

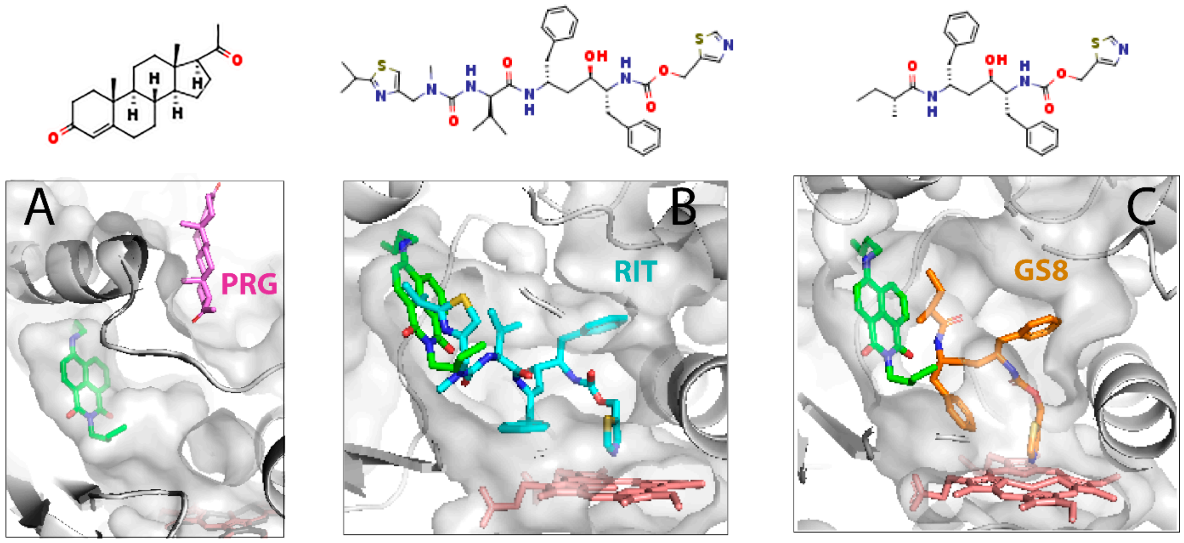

Figure S2. Superposition of fluorol-bound CYP3A4 with other ligand-bound forms: progesterone, ritonavir and GS8 (**A**-**C**, respectively). Fluorol is depicted in green sticks, progesterone (PRG) in magenta (5A1R structure), ritonavir (RIT) in cyan (3NXU structure), and GS8 in orange (4K9X structure). Progesterone docks to the remote peripheral area and should not affect the intra-channel binding of fluorol. For GS8, only minor adjustments in the flexible end-moiety are needed to accommodate fluorol in the substrate channel. The bulkier tail of ritonavir occupies most of the substrate channel, which could preclude association of fluorol or force it to dock at the entrance of the channel.

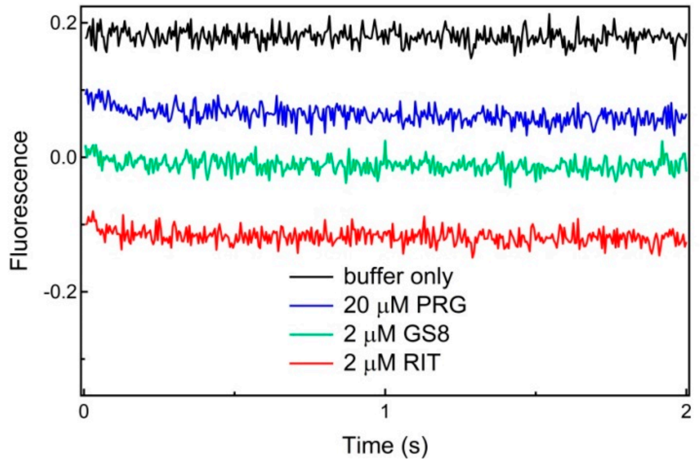

Figure S3. Control kinetic traces recorded after mixing of 2 μM fluorol with a buffer in the absence or presence of progesterone (PRG), GS8 or ritonavir (RIT). Traces were offset to demonstrate the lack of feasible fluorescence changes within the studied time interval when CYP3A4 was absent. The y-axis range is the same as in Figure 6E, F in the main text.

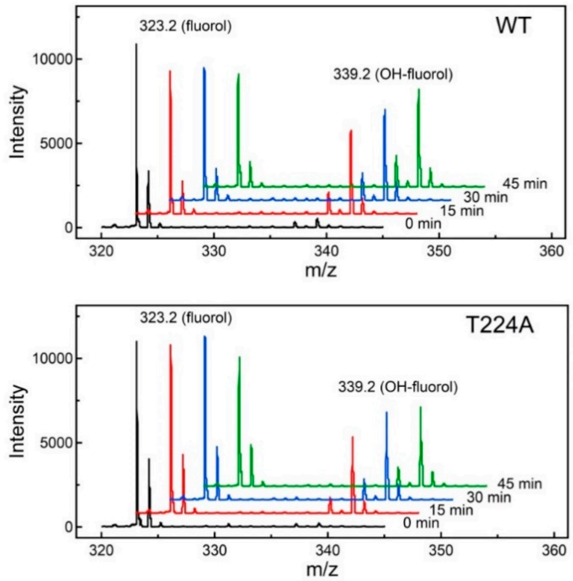

Figure S4. Time course of the fluorol oxidation reaction catalyzed by WT and T224A CYP3A4 (upper and middle panels, respectively) in a soluble reconstituted system with cytochrome P450 reductase. Time dependent changes in the substrate:product ratios are shown in Figure 7B of the main text.

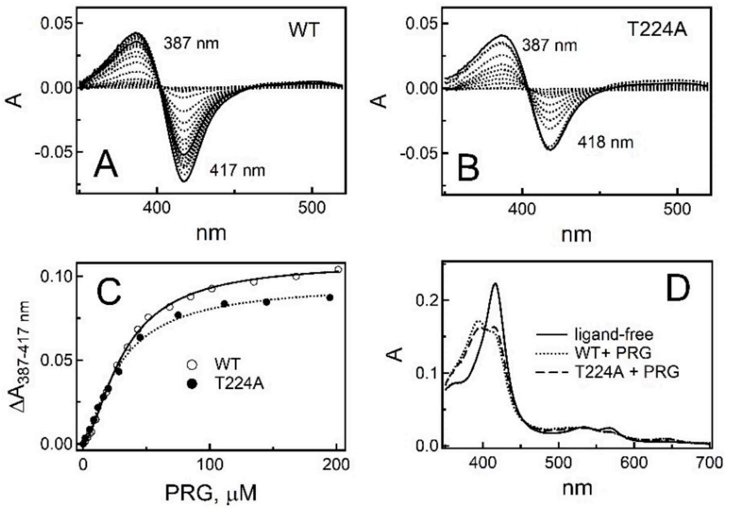

Figure S5. Effect of the T224A mutation on the binding of progesterone (PRG). **A** and **B**, Difference absorbance spectra recorded during equilibrium titrations of 2 μM WT and T224A CYP3A4, respectively, with PRG. **C**, Titration plots with sigmoidal fittings. The derived PRG concentration at half saturation (S_{50}) and Hill coefficient (n_H) were 32±2 μM and 1.56 for WT and 25±4 μM and 1.37 for T224A CYP3A4. **D**, Spectra of ligand-free and PRG-bound CYP3A4 recorded at the end of titration experiments. Elimination of Thr224 leads to a 9% decrease in the high-spin content in the PRG-bound CYP3A4: from 71% in WT to 62% in the T224A mutant.
